# Initial joint stability affects the outcome after conservative treatment of simple elbow dislocations: a retrospective study

**DOI:** 10.1186/s13018-015-0273-x

**Published:** 2015-08-20

**Authors:** Marc Schnetzke, Sara Aytac, Stefan Studier-Fischer, Paul-Alfred Grützner, Thorsten Guehring

**Affiliations:** Berufsgenossenschaftliche Unfallklinik Ludwigshafen, Abteilung für Unfallchirurgie und Orthopädie, Ludwig Guttmann Straße 13, 67071 Ludwigshafen am Rhein, Germany

**Keywords:** Elbow dislocation, Fluoroscopy, Stress test, Elbow instability, Drop sign, Joint incongruence

## Abstract

**Background:**

Conservative treatment of simple elbow dislocations can lead to complications such as persisting pain and restricted joint mobility. The current aim was to identify patients with deteriorated outcome after conservative treatment and to investigate a possible association with initial joint (in)stability.

**Methods:**

Sixty-eight patients (mean age 37.1 ± 17.2 years) with simple elbow dislocations received conservative treatment. After closed reduction, joint stability was tested by varus and valgus stress under fluoroscopy. According to the findings under fluoroscopy, three different groups of instability could be identified: (1) slight instability (joint angulation <10°; *n* = 49), (2) moderate instability (angulation ≥10°; *n* = 19) and (3) gross instability. Patients with gross instability (re-dislocation under stability testing) were treated with primary surgical ligament repair and therefore excluded from this study. Additionally, MRIs and radiographs were analysed regarding warning signs of instability such as the drop sign and joint incongruence. Main outcome parameters were the Mayo Elbow Performance Score (MEPS), range of motion (ROM), complications and revision rates.

**Results:**

After 40.7 ± 20.4 months, the overall MEPS was excellent (94.2 ± 11.3) with a trend of slightly worse clinical results in group 2 (95.8 ± 9.0 vs. 90.0 ± 15.2 points; *p* = 0.154). In group 1, significantly more patients achieved an excellent result regarding the MEPS scoring system (77.6 vs. 52.6 %; *p* = 0.043) and elbow extension was significantly worse in group 2 (5.3 ± 9.9° vs. 1.4 ± 3.0°; *p* = 0.015). Seven treatment complications occurred in group 2 (36.8 %) compared with two in group 1 (4.1 %, *p* < 0.0001). Six patients (8.8 %) needed secondary surgery with an 8.4-fold higher risk for revision surgery in group 2 (*p* = 0.007). The presence of a positive drop sign or joint incongruence led to higher odds ratio (OR) for complications (OR = 15.9) and revision surgery (OR = 10.3).

**Conclusions:**

This study demonstrates that patients with moderate joint instability after simple elbow dislocation have a significantly worse clinical outcome, more complications and a higher need for secondary revision surgery following conservative treatment compared to patients with slight elbow instability.

## Background

The elbow is the second most commonly dislocated major joint [1]. It can be classified as simple or complex, dependent on the injury pattern, and simple dislocations are defined by the absence of concomitant fractures [2].

A relative consensus exists in favour of a conservative treatment of simple elbow dislocations in the absence of any tendency of re-dislocation within the functional arc of the joint [3, 4]. It is also a fact that unstable simple elbow dislocations with re-dislocation under stability testing benefit from early surgical ligament repair rather than by conservative treatment [5–7].

However, the outcome after conservative treatment of simple elbow dislocations is not always satisfactory. Anakwe et al. reported about complications such as persisting pain and stiffness in more than half of the patients in the long-term follow-up of 110 patients treated with closed management [8] and concluded that simple elbow dislocations may not be entirely benign. Similarly, others reported comparable results with a restriction of range of motion (ROM) and persisting pain in 35–80 % and chronic instability in 15–35 % in long-term follow-up, which leads to an inferior clinical outcome [9–12, 4, 8].

A satisfying clinical outcome may rely on the initial joint stability after joint reduction and early functional rehabilitation. This is based on the finding that in the remaining instability of the elbow during rehabilitation, a posttraumatic arthrofibrosis can develop [13–15]. Thus, a chronic instability should be avoided by all means, as then a secondary augmentation with triceps tendon or hamstring tendons must be considered [16, 17].

Recently, Hackl et al. published a comprehensive review regarding the treatment of simple elbow dislocations. The authors concluded that there is still lack of evidence for the individual treatment decision of simple elbow dislocations [18].

Thus, the aim of this study was to identify trauma-related risk factors for the development of complications associated with a conservative treatment after simple elbow dislocations. In particular, the effect of initial joint stability under fluoroscopy on the clinical outcome was investigated. Secondary, the predictive value of warning signs of instability such as the drop sign and joint incongruence was analysed.

## Methods

### Study population

This retrospective level III study was done in agreement with the local ethical review committee (No. 837.084.14 (9323-F)). Between January 2009 and November 2013, 76 patients received conservative treatment after simple elbow dislocation at a level 1 trauma centre.

Eight patients were lost to follow-up due to change of residency. A total of 68 patients (89.5 %) with a mean age of 37.1 ± 17.2 years (18 to 69) were included in this study. Forty patients were males (58.8 %) and 28 were females (41.2 %).

Patients with simple elbow dislocations with ligament or capsular injuries and avulsion fractures of the coronoid (type I) according to Regan and Morrey were included. This definition of simple elbow dislocations is in accordance with recent studies [6, 5]. Patients with associated articular fractures of the radial head, the olecranon or coronoid fractures type II and III, or relevant chondral lesions were excluded. Patients under the age of 18 and patients with known previous elbow injuries as well as patients with severe comorbidities such as autoimmune disease, malignancies or heart insufficiency were also excluded.

### Stability testing under fluoroscopy

After immediate joint reduction under analgesia or anaesthesia successful joint reduction was confirmed by X-rays. Immediately after closed reduction, joint stability was tested under fluoroscopy in full extension, 30° of flexion, pronation and supination, and varus and valgus stress, respectively. According to the stability testing under fluoroscopy, three different groups of elbow instability could be identified: (1) slight instability, (2) moderate instability and (3) gross instability.

Simple elbow dislocations with gross instability re-dislocated under stability testing and received primary surgical ligament repair. Therefore, this group of patients were excluded from this study. Patients that did not re-dislocate under fluoroscopy were assigned to two different groups according to their initial joint stability under fluoroscopy. The elbow was rated as slightly instable in case of less than 10° joint angulation on the medial and/or lateral side (group 1) and as moderate instable in case of more than 10° of joint angulation (group 2). Patients with gross instability did not allow quantitative measurements of joint angulation as a result of early dislocation during stability testing (group 3, excluded).

This “threshold” of joint stability was adopted from cadaver experiments by Olsen et al. as a stepwise transection of the lateral and medial collateral ligament (LCL and MCL) led to an increased joint laxity of approximately 11.7° [19, 20].

All patients with slight elbow instability received a conservative treatment with closed reduction and functional rehabilitation. Patients with moderate instability were treated either conservatively with closed management or surgically with primary surgical ligament repair. Each treatment decision was taken individually dependent on factors such as age, involved side, state of activity, profession, and patient’s individual preference. In the current study, only patients with conservative treatment were included. The inclusion and exclusion criteria are shown in Table 1.

**Table 1:** Inclusion and exclusion criteria

Inclusion criteria	Exclusion criteria
Age >18 years	Previous elbow injury
Informed consent	Severe comorbidities (e.g. malignancy, autoimmune disease)
Conservative treatment after simple elbow dislocation	Primary surgical treatment after simple elbow dislocation

### Rehabilitation protocol

Irrespective of the joint stability, all patients underwent temporary plaster immobilization in a posterior splint at 90° of elbow flexion for 2 weeks. According to the rehabilitation protocol, functional treatment began within 7 days at the latest after trauma with exercises in a pain-free range of motion under avoidance of forearm rotation in the first 6 weeks.

### Injury pattern

All patients had a posterior elbow dislocation. Seventeen patients (25.0 %) had a capsular injury or a coronoid fracture type I. The left elbow was injured in 46 patients (67.6%) and the right elbow in 22 patients (32.4%). Details of injury patterns with variation of treatment groups according to the MRI, radiographs and/or fluoroscopy are shown in Table 2.

**Table 2:** Distribution of injury pattern according to MRI and fluoroscopic findings with variation of treatment groups

Injured ligament	Group 1 (*n* = 49)	Group 2 (*n* = 19)	Total (*n* = 68)
Lateral	26 (53.1 %)	4 (21.1 %)	30 (44.1 %)
Medial	15 (30.6 %)	9 (47.4 %)	24 (35.3 %)
Medial and lateral	1 (2.0 %)	6 (31.6 %)	7 (10.3 %)
Partial tear	7 (14.3 %)	0	7 (10.3 %)
Capsular injury/Regan and Morrey I	13 (26.5 %)	4 (21.1 %)	17 (25 %)

### Radiographic analysis

**Fig. 1:**
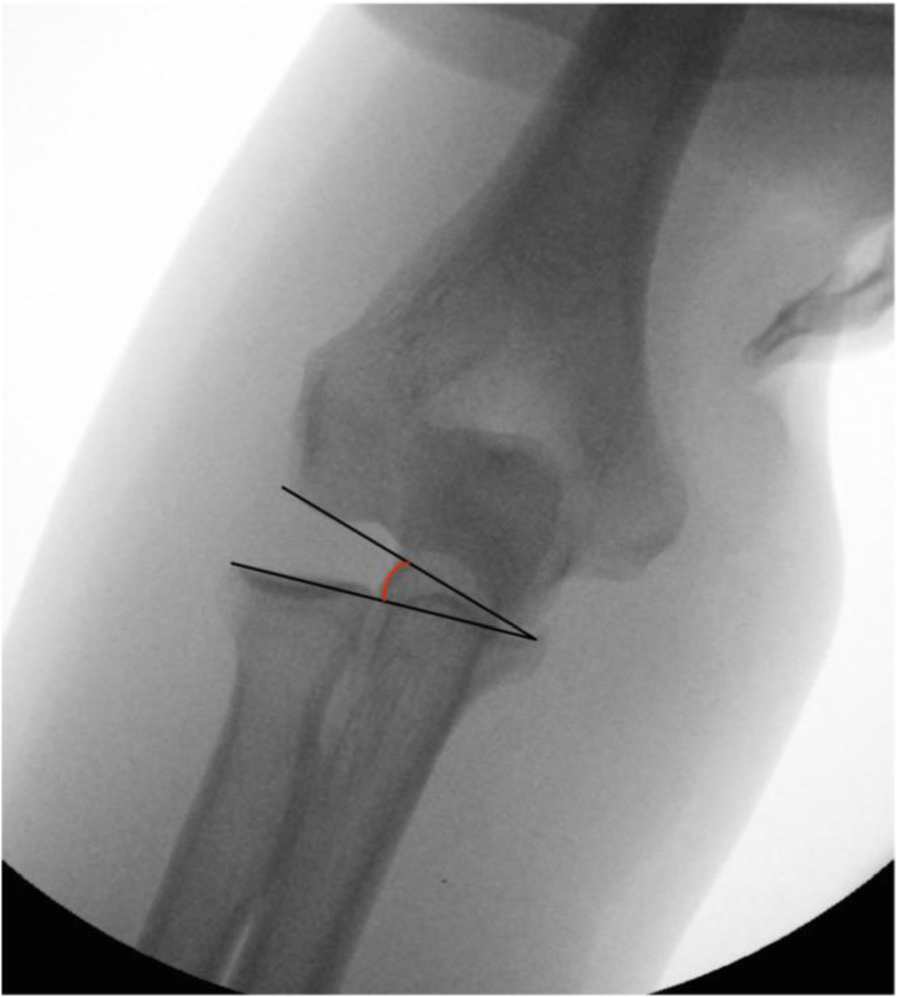
Stability testing in full extension and supination under fluoroscopy: lateral joint angulation in stress valgus position (alpha) is 14.1°

To determine joint instability under fluoroscopy, the medial or lateral joint angulation (alpha) between the distal humeral joint line and the proximal ulno-radial joint line was measured. This procedure was done in maximal varus and valgus stress as described before. The measurement of joint angulation was done in antero-posterior projection, and only small differences in rotation were accepted. Figure 1 illustrates the measurement procedure.

In addition to the stability testing under fluoroscopy, MRIs were recommended in all patients to detect intra-articular pathologies (i.e. chondral lesions). In 22 patients (32.4 %), MRIs were not available because the further treatment was done elsewhere after the patient has been discharged from inpatient treatment. Finally, MRIs were available in 46 patients (67.6 %).

**Fig. 2:**
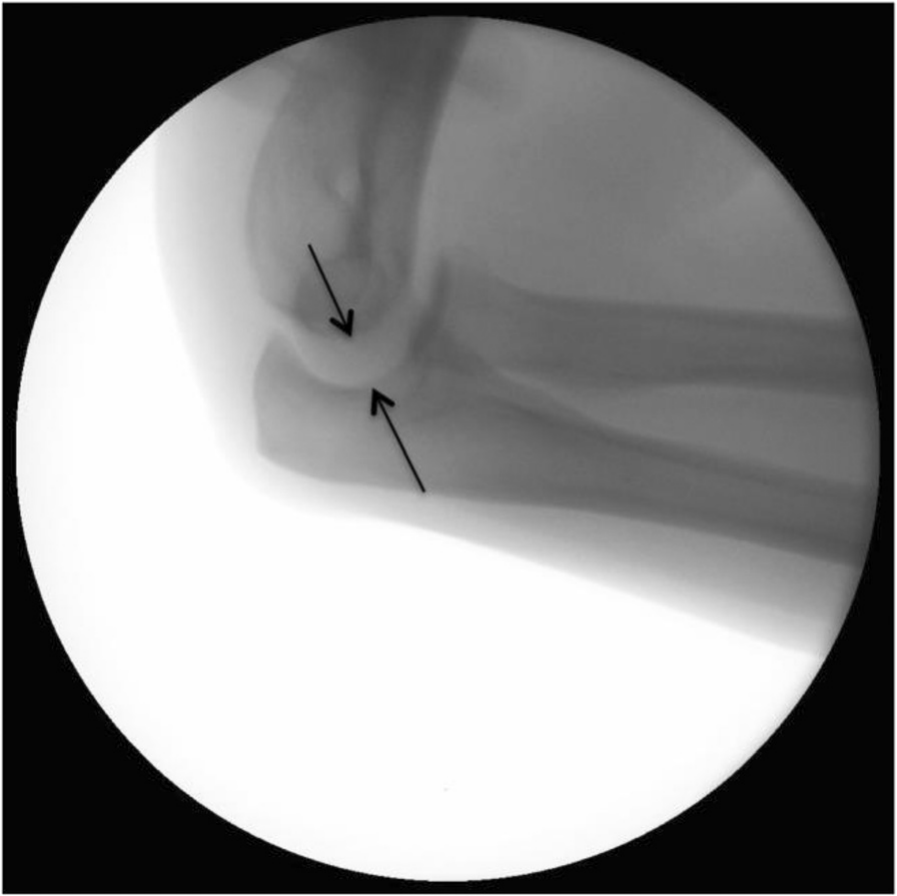
Increased humero-ulnar distance of 9 mm (positive drop sign) in lateral view of the elbow (*black arrows*)

**Fig. 3:**
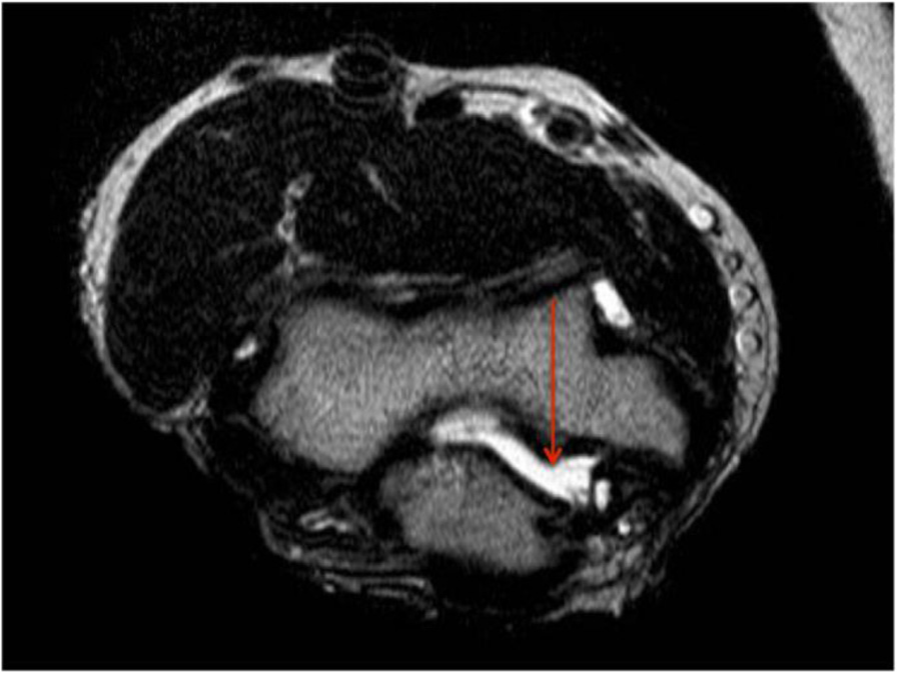
Complete tear of the MCL with humero-ulnar joint incongruence at the medial side (*red arrow*) in axial view in MRI

**Fig. 4:**
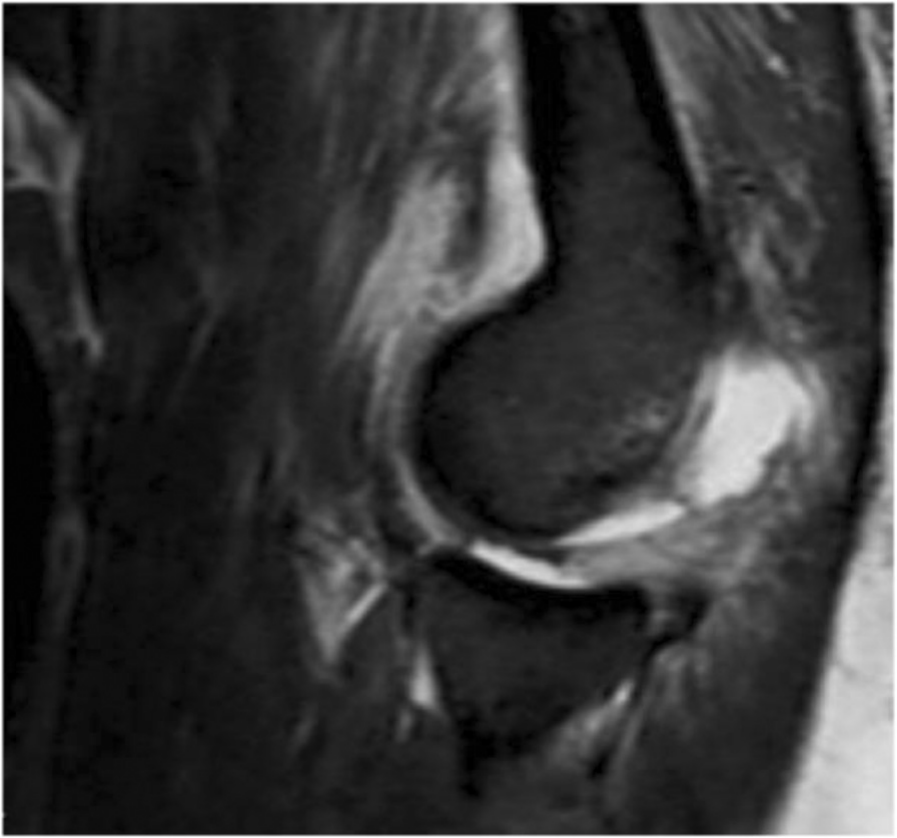
Complete tear of the LCL with posterior subluxation of the radial head in sagittal view in MRI

**Fig. 5:**
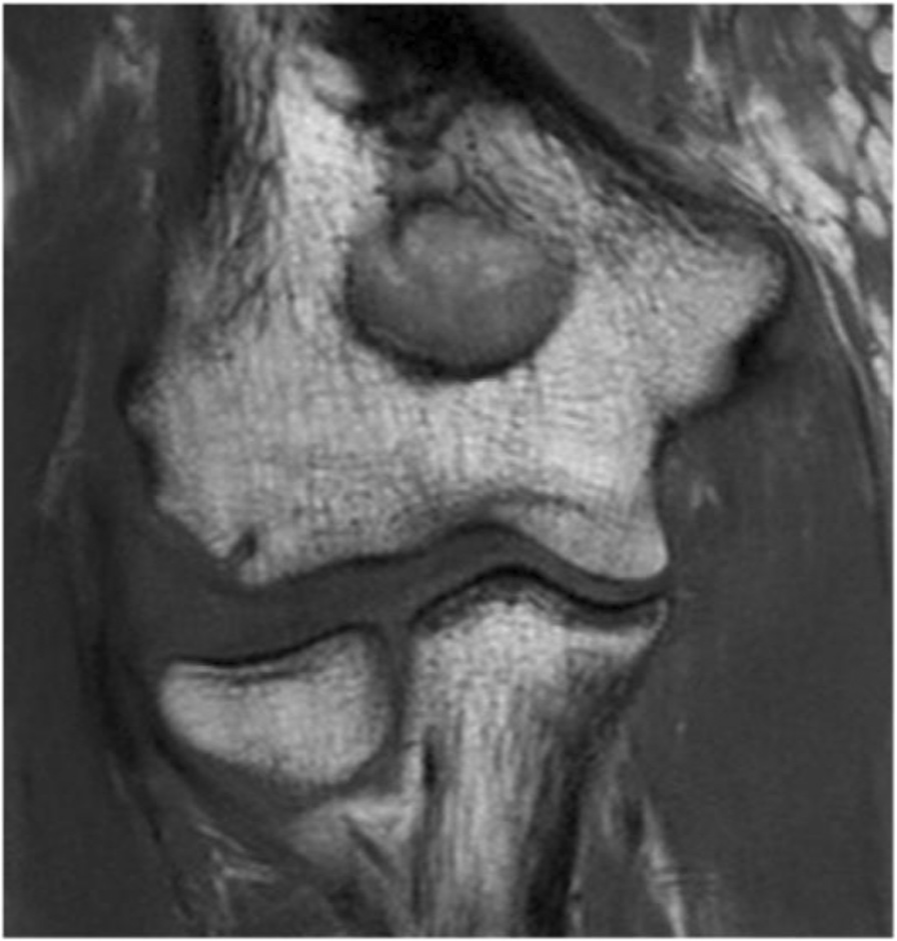
Complete tear of the LCL with radio-humeral joint incongruence in coronal view in MRI

Beyond the varus and valgus stress testing under fluoroscopy, the joint (in)stability was determined by two other possible warning signs of instability: the drop sign [21] and joint incongruence [22]. As a sign of severe joint instability, the drop sign is defined by a humero-ulnar distance of at least 4 mm (Fig. 2) [21]. The drop sign was measured at the first lateral X-ray view of the elbow taken after immediate joint reduction [23]. The measurement procedure was done in accordance to the recommendations of Coonrad et al. with the trochlear sulcus as the proximal portion and the centre of the articular surface of the olecranon as the distal portion of the humero-ulnar measurement [21]. The joint incongruence was determined in MRIs by a noncongruence of the humero-ulnar and humero-radial joint lines by an independent and blinded radiologist (Figs. 3, 4 and 5). Additionally, the correlation of the MRIs with the fluoroscopic findings was evaluated.

It should be noted that the MRIs were only analysed in full joint extension because in this position, the collateral ligaments are tensioned [24], which allows an appropriate evaluation of the ligaments. Therefore, the analysis of joint incongruence and a correlation of the MRI with the fluoroscopic findings could only be done in 32 patients (47.1 %).

### Clinical outcome parameters with mid-term follow up

At follow-up, the clinical outcome was determined by the Mayo Elbow Performance Score (MEPS) [25], the visual analogue pain scale (VAS), elbow stability and ROM. The flexion and extension deficit of the elbow was tested comparing the injured with the healthy side. Trauma- and treatment-associated complications (restriction of ROM >30°, chronic instability), secondary surgeries and return to previous work were determined.

### Statistics

Data were processed with SPSS 22.0. Mean and ranges were calculated for continuous, mean and median for ordinal variables. The primary outcome parameter was the MEPS. Differences between both treatment groups were analysed using the Student´s T. A two-tailed *p* value of <0.05 was considered to show a significant difference. Pearson chi-square test was used in the analysis of contingency tables.

## Results

### Clinical outcome

Sixty-eight patients were followed for a mean period of 40.7 ± 20.4 months. At recent follow-up, the overall MEPS was excellent with 94.2 ± 11.3 points and average VAS numbers were 0.8 ± 1.6. Fifty-two patients (76.5 %) had no restriction of ROM, mean extension deficit was 2.5° ± 6.0° and mean flexion deficit was 1.5° ± 5.2°. Three employees (5.4 %) had to change work after rehabilitation, and one patient (1.8 %, 62 years) did not manage to return to previous work.

According to the initial stability testing under fluoroscopy, 49 patients showed slight elbow instability and were referred to group 1 (72.1 %) and 19 patients showed moderate elbow instability and were assigned to group 2 (27.9 %). Age, gender and affected side were equally distributed in both groups (Table 3). The subgroup analysis revealed that significantly more patients in group 1 achieved an excellent outcome regarding to the scoring system of the MEPS (77.6 vs. 52.6 %; *p* = 0.043), the average MEPS showed a trend of worse outcome in group 2 without significant difference (95.8 ± 9.0 vs. 90.0 ± 15.2 points; *p* = 0.154). All patients achieved an arc of motion of 100° at final follow-up. Detailed analysis of the ROM revealed that the extension was significantly worse in group 2 (5.3° ± 9.9° vs. 1.4° ± 3.0°; *p* = 0.015), whereas the flexion was comparable between both groups (*p* = 0.223). The detailed analysis for the terms of MEPS, ROM and chronic instability with variation of both treatment groups is shown in Table 4.

**Table 3:** Basis demographic data with variation of treatment groups

	Group 1 (*n* = 49)	Group 2 (*n* = 19)	*p* value
Age [years]	36.3 ± 17.4	39.4 ± 16.7	0.509
Gender [%]			0.535
Female	19 (38.8)	9 (47.4)	
Male	30 (61.2)	10 (52.6)	
Affected side			0.221
Right	18 (36.7)	4 (21.1)	
Left	31 (63.3)	15 (78.9)

**Table 4:** Clinical parameters at final follow up with variation of treatment groups

Parameter	Group 1 (*n* = 49)	Group 2 (*n* = 19)	*p* value
MEPS [pts]	95.8 ± 9.0	90.0 ± 15.2	0.154
Subgroup analysis [%]			
Excellent	38 (77.6)	10 (52.6)	*0.043*
Good	8 (16.3)	7 (36.8)	0.275
Fair	3 (6.1)	1 (5.2)	
Poor	0	1 (5.2)	
VAS	0.6 ± 1.5	1.2 ± 1.8	0.228
Unrestricted ROM [%]	40 (81.6)	12 (63.2)	0.195
Complete ROM [°]	137.1 ± 8.0	133.5 ± 11.6	0.250
Extension deficit [°]	1.4 ± 3.0	5.3 ± 9.9	*0.015*
Flexion deficit [°]	1.4 ± 5.7	1.8 ± 4.0	0.223
Chronic instability [%]	0	3 (15.8)^a^	

^a^Two patients had lateral ligament reconstruction for chronic instability and had a stable elbow joint at final follow-up

**Fig. 6:**
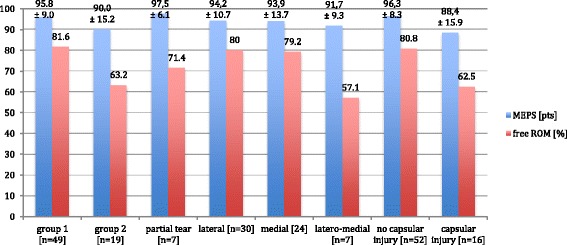
Detailed mean value of MEPS with standard deviation and [%] of unrestricted ROM at follow-up

The evaluation of the different injury types with disregard of the grade of instability revealed no significant difference concerning the main outcome parameter MEPS and ROM (*p* > 0.05 between all groups; for detailed results, see Fig. 6). Additional capsular or coronoid type I injuries were associated with a trend of worse clinical outcome according to the MEPS (*p* = 0.076).

### Radiographic results

Varus and valgus stability testing under fluoroscopy was done in all patients. Mean joint angulation in patients with lateral ligament injury was 5.0° ± 4.1° and in medial ligament injury 9.5° ± 4.8°. In patients with radio-ulnar ligament injury, radial joint angulation was 7.8° ± 3.5° and the ulnar joint angulation 8.7° ± 6.7°.

After assignment to the different groups of instability, patients with moderate elbow instability had a mean joint angulation of 11.5° ± 1.8° at the lateral side and 13.7° ± 2.3° at the medial side. Nine patients of group 2 (50 %) and none in group 1 had a positive drop sign with a mean humero-ulnar distance of 5.5 ± 3.1 mm.

Thirty-two MRIs could be analysed (47.1 %). Mean duration between trauma and the MRI was 6.6 ± 6.2 days. In group 2, MRIs of ten patients could be evaluated and in three MRIs (30 %), a joint incongruence was described. In addition, all three patients with joint incongruence in MRI had a positive drop sign. In group 1, MRIs of 22 patients were analysed and no joint incongruence was found.

**Fig. 7:**
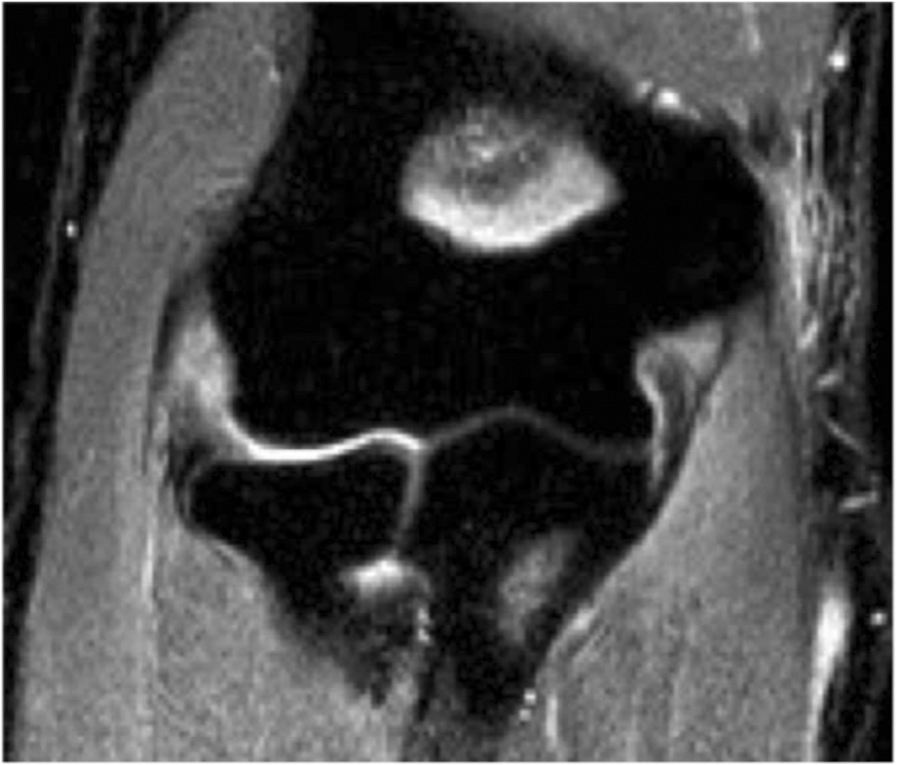
Example of a complete LCL tear and partial MCL tear in MRI

**Fig. 8:**
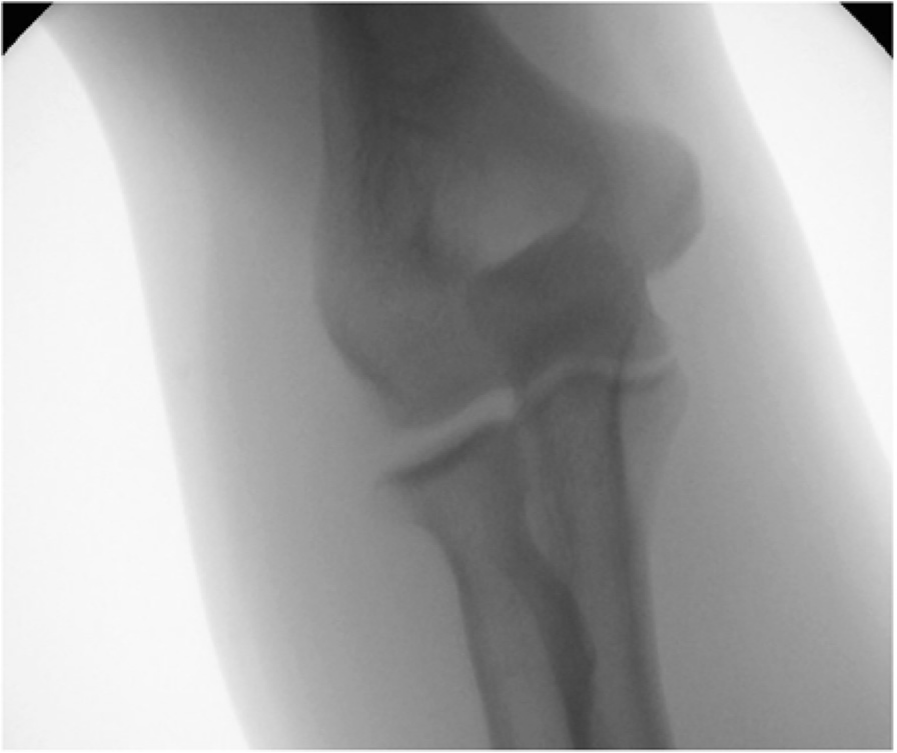
Fluoroscopy in full extension and supination detected no lateral joint instability in varus stress position (2° of lateral joint angulation)

The comparison of fluoroscopic and MRI findings showed a relatively reliable agreement of diagnosed MRI and fluoroscopic injury pattern in 25 of 32 patients (78.1 %), i.e. patients with lateral ligament rupture in MRIs had similarly a lateral joint instability under fluoroscopy, and vice versa. However, in four patients (12.5 %), the elbow was stable under fluoroscopy (joint angulation of <5°) although MRI findings indicated a relevant ligament injury (Figs. 7 and 8). In three patients (9.4 %), the elbow had a higher joint angulation (>5°) even if only a partial ligament injury was seen in the MRI.

### Complications and revisions

Eight patients (11.8 %) developed a total of nine complications. Seven complications occurred in group 2 (36.8 %) compared to only two complications in group 1 (4.1 %), demonstrating a significantly higher occurrence in group 2 (*p* < 0.0001). Patients with moderate elbow instability had an 8.4-fold higher odds ratio (OR) for a secondary surgery (*p* = 0.007) and 10.8-fold higher risk for complications, respectively (*p* = 0.002). Details for complications and secondary surgeries are shown in Table 5.

**Table 5:** Complications and secondary surgeries with variation of treatment groups and OR

Parameter	Group 1 (*n* = 49)	Group 2 (*n* = 19)	Odds ratio
Complications (total)	2 (4.1 %)	7 (36.8 %)	10.8
Restriction of ROM >30°	2	4	
Chronic instability	0	3	
Secondary surgery (total)	2 (4.1 %)	4 (21.1 %)	8.4
Arthroscopic release	2	2	
Ligament reconstruction	0	2	

The presence of a positive drop sign or joint incongruence in MRI led to significantly higher risk for the development of complications or the need for secondary surgery (15.9- and 10.3-fold, respectively).

## Discussion

Simple elbow dislocations are currently predominantly treated by conservative treatment with often satisfying clinical outcome [26, 4]. However, some patients develop residual pain, elbow stiffness or chronic elbow instability, which is then difficult to treat [9, 8]. Here, we show differential results after conservative treatment of both elbows with slight *and* moderate instability following simple elbow dislocation and found good results regarding MEPS (94.2 ± 11.3 points), VAS (0.8 ± 1.6) and unrestricted range of motion in 76.5 %, in agreement with other reports [26, 3]. It should be noted that patients with gross instability with re-dislocation under stability testing were excluded in this study. Those patients received primary surgical ligament repair.

In patients with slight elbow instability (joint angulation <10°), a conservative treatment with early functional treatment is the treatment of choice and the current study showed an excellent treatment outcome with an average MEPS of 95.8 ± 9.0 points. Similarly, 19 patients with moderate elbow instability (joint angulation ≥10°) underwent conservative treatment, and despite the fact that these patients finally also had a good outcome (MEPS of 90.0 ± 15.2 points), the subgroup analysis revealed that significantly more patients in group 1 achieved an excellent result according to the scoring system of the MEPS (77.6 vs. 52.6 %; *p* = 0.043). Moreover, detailed analysis of the ROM showed that the elbow extension was significantly worse in group 2 (5.3° ± 9.9° vs. 1.4° ± 3.0°; *p* = 0.015). In other words, 36.8 % of patients with moderate instability did not achieve an unrestricted ROM and 47.4 % of those patients could not achieve an excellent outcome according to the MEPS scoring system under conservative treatment. This is in agreement with other reports [8–11]. It should be further noted that without secondary surgery (4 of 19 patients in group 2, 21 %), the clinical outcome might be considerably worse in group 2 with lower MEPS points due to the limited ROM or persisting joint instability.

The current study also demonstrated that patients with initially moderate elbow instability developed significantly more complications and had a higher need for a revision surgery. Seven patients (36.8 %) with moderate instability developed complications, leading to a significantly higher relative risk for complications (OR = 10.8) and secondary surgery (OR = 8.4) compared to patients with slight instability. Conversely in group 1, only two patients (4.1 %) showed complications and no patient developed a chronic instability. The presence of a warning sign of instability (drop sign or joint incongruence) led to even higher OR for complications (OR = 15.9) and revision surgery (OR= 10.3).

A variety of diagnostic tools should enable the physician to treat the elbow dislocation in the most appropriate way to avoid posttraumatic sequelae. MRIs allow to detect injured structures with a high sensitivity [22], though it does not enable to quantify instability. In contrast, fluoroscopy is a good diagnostic tool for detection of joint instability [27, 14]. In accordance to our current results, Eygendaal et al. compared their findings on MRI and fluoroscopy in chronic instability and found congruency in 10 of 13 patients (76.9 %), whereas 3 patients were unstable without findings in the MRI [27]. In our study, MRIs and fluoroscopy were in agreement in 25 of 32 patients (78.1 %). As a limitation of this study, MRIs were only done in 32 out of 68 patients (47.1 %). Nevertheless, these findings support the concept that MRI and fluoroscopy provide different but mandatory information, and fluoroscopy allows to provide additional important information about functional joint stability rather than injury pattern alone. In conclusion, the combination of both may thus help to guide between the appropriate treatment options.

Our strategy of treatment of patients with simple elbow dislocations is in agreement with the literature. A simple stable elbow dislocation can be treated by functional extension/flexion exercises with brace or bandage early after injury, and there is evidence that a functional treatment leads to less joint stiffness and better joint mobility, compared to plaster immobilisation [4, 28], particularly if the immobilisation takes longer than 2 weeks [3]. Mehlhoff et al reported that a prolonged immobilisation decreased the outcome in 52 patients with simple dislocations, with a 15 % complication rate showing a flexion contracture over 30° [10]. Eygendaal et al. reported about detailed follow-up analysis of 50 patients with posterolateral dislocation of the elbow treated by closed reduction and conservative treatment [11]. A valgus radiographic stress X-ray study was performed on every patient and revealed that almost 50 % (24 of 50) of the patients had a clear radiographic evidence of valgus instability with deterioration of clinical long-term results.

There is only one randomized controlled study by Josefsson et al. that compared ligament repair with conservative treatment in 28 simple elbow dislocations and did not find evidence that ligament repair was beneficial [29]. However, in this study, a relative high rate of chronic instability (14.7 %) and loss of extension remained after conservative treatment, as compared to the here found instability rate of 2.9 % after conservative treatment. The loss of extension was 18° ± 15° in surgical treatment group and 10° ± 14° in the nonsurgical group. In general, 3°–8° loss of extension can be expected [26]. The surgical treatment and the understanding of the elbow anatomy and kinematics have improved a lot; therefore, the study of Josefsson et al. is more of historical interest. Recent studies revealed excellent results after primary surgical treatment of unstable simple elbow dislocations [6, 7, 30–32].

The current study results thus support the notion that simple elbow dislocation injuries indeed may not be entirely benign, and the rate of residual pain and elbow stiffness might be underestimated [8], as supported by a relative high rate of persisting instabilities. In this study, a group of patients with persisting complaints after conservative treatment could be identified. Simple elbow dislocations with moderate instability under varus and valgus stress test under fluoroscopy had significantly more complications and a significantly worse clinical outcome compared to patients with slight elbow instability. Therefore, in younger and active patients with moderate elbow instability under stability testing, primary surgical treatment should be considered to avoid such complications and decrease the need for secondary surgery [7, 6, 5]. The results of this study emphasize the importance of an initial joint stability testing under fluoroscopy for the individual treatment decision. The combination with MRIs and radiographs may be an appropriate diagnostic tool to distinguish between elbows that can be treated with closed management alone or with primary surgical ligament repair.

This study has several limitations. We report about a series of patients with traumatic simple elbow dislocations. There was no randomisation for conservative treatment. With a mean follow-up of 40.7 months, no information can be provided for long-term follow-up. Evaluation of the data was retrospective, and a power analysis was not performed. MEPS was used as main outcome parameter, which is one of the most commonly used physician-based elbow rating systems. As a limitation, MEPS does not take both patient’s and physician’s perspective into account [25].

## Conclusion

The current results demonstrated a differential outcome after conservative treatment of simple elbow dislocation. Patients with moderate elbow instability (joint angulation of more than 10°) showed a significantly higher risk for complications and secondary surgery compared to patients with slight elbow instability. Furthermore, clinical outcome was significantly worse regarding elbow extension and number of patients achieving an excellent outcome regarding the MEPS scoring system. This study revealed, that initial stability testing under fluoroscopy is important to guide management to decide whether a simple elbow dislocation can be treated conservatively or whether primary surgical treatment options should be considered to avoid posttraumatic sequelae. Further studies will be required to confirm these findings and will answer the question, whether particularly patients with moderate elbow instability under fluoroscopy may benefit from early surgical repair.

## Abbreviations

MEPS: Mayo Elbow Performance Score
ROM: range of motion
OR: odds ratio
MRI: magnetic resonance imagine
MCL: medial collateral ligament
LCL: lateral collateral ligament
VAS: visual analogue pain scale
